# Successful development of methodology for detection of hapten-specific contact hypersensitivity (CHS) memory in swine

**DOI:** 10.1371/journal.pone.0223483

**Published:** 2019-10-09

**Authors:** E. J. Putz, A. M. Putz, A. Boettcher, S. Charley, M. Sauer, M. Palmer, R. Phillips, J. Hostetter, C. L. Loving, J. E. Cunnick, C. K. Tuggle

**Affiliations:** 1 Iowa State University, Department of Animal Science, Ames, IA, United States of America; 2 Iowa State University College of Veterinary Medicine, Department of Veterinary Pathology Science, Ames, IA, United States of America; 3 USDA-ARS-National Animal Disease Center, Infectious Bacterial Diseases of Livestock Research Unit, Ames, IA, United States of America; 4 USDA-ARS-National Animal Disease Center, Food Safety and Enteric Pathogens Research Unit, Ames, IA, United States of America; Tulane National Primate Research Center, UNITED STATES

## Abstract

Hapten contact hypersensitivity (CHS) elicits a well-documented inflammation response that can be used to illustrate training of immune cells through hapten-specific CHS memory. The education of hapten-specific memory T cells has been well-established, recent research in mice has expanded the “adaptive” characteristic of a memory response from solely a function of the adaptive immune system, to innate cells as well. To test whether similar responses are seen in a non-rodent model, we used hapten-specific CHS to measure the ear inflammation response of outbred pigs to dinitrofluorobenzene (DNFB), oxazolone (OXA), or vehicle controls. We adapted mouse innate memory literature protocols to the domestic pig model. Animals were challenged up to 32 days post initial sensitization exposure to the hapten, and specific ear swelling responses to this challenge were significant for 7, 21, and 32 days post-sensitization. We established hapten-specific CHS memory exists in a non-rodent model. We also developed a successful protocol for demonstrating these CHS responses in a porcine system.

## Introduction

The contact hypersensitivity (CHS) memory response to the haptens dinitrofluorobenzene (DNFB) or oxazolone (OXA), typically driven by T cells, is well-documented [1–4]. This memory can be illustrated by the ability to form specific, long-lived memory responses to hapten-modified proteins, the re-exposure of which triggers a delayed but specific CHS response. T cell hapten responses are initiated by antigen presenting cells (APCs) such as Langerhans or dendritic cells that, upon activation, migrate to draining lymph nodes from the site of challenge [5]. In lymph nodes, APC presentation of haptenized antigen to naïve T cells facilitates clonal expansion to create long lived, hapten-specific CD4+ and CD8+ T cells [6]. Unlike effector T cells, Natural Killer (NK) cells are cytotoxic innate lymphocytes that target cells lacking or not displaying ‘self’ Major Histocompatibility Complex I (MHC I) [7]. Recently, the classically adaptive characteristic of immune memory has been expanded to certain innate cells, including monocytes [8] and NK cells [2, 9–11]. Several studies used similar CHS challenges in mice to show innate immune memory can be driven by NK cells, with an emphasis on liver resident NK cell populations [2, 9, 10, 12].

Haptens have previously been used to elicit inflammatory responses in swine. Both DNFB and OXA haptens have been shown to react in a porcine model of allergic contact dermatitis [13, 14]. However, to our knowledge, no work has been done to confirm hapten-specific CHS memory exists in the swine model.

This study seeks to investigate the parameters of hapten-specific CHS memory responses in a large animal model. The pig is more similar to the human immunologically and physiologically, with a closer “immunome” [15] to humans than is the mouse [16]. The pig may be an advantageous model to study relationships between adaptive and innate systems [17] as well as the specific mechanism of innate memory. Developing tools to manipulate porcine innate memory (if present) could be relevant to commercial swine health in vaccine development as well as model human therapies. However, to facilitate development of the pig as a large animal model for such studies, a CHS memory protocol for swine is lacking; we establish such a protocol herein.

## Materials and methods

All animal experiments were approved by the Iowa State University Institutional Animal Care and Use Committee (IACUC).

### Animals

Commercial outbred 15–30 kg finishing pigs were purchased from the Iowa State University Swine Nutrition Farm, where they were individually housed for all trials. Animals were maintained in traditional commercial swine stalls, exposed to typical 12 hour light/dark cycles, and fed ad lib food and water. For sensitization studies: 20 pigs were used 5 day sensitization trial, 20 pigs were used for the 32 day sensitization trial, 36 pigs were used for the 21 day sensitization trial, and 38 pigs were used for the 7 day sensitization trial.

### Sensitization and challenge

For 5 or 32 day sensitization experimental trials involving intradermal injections of haptens (described below, see S1 Fig), pigs were sedated with intramuscular telazol (100mg/mL)/ketamine(100mg/mL)/xylazine(100mg/mL) (TKX)(50 μl/kg) injection for sensitization and challenge procedures. For 7 and 21 day sensitization trials, topical application, which is less affected by body movement of the pig, lower TKX dosages (25 μl/kg) were used. Pigs were housed individually at the Iowa State University Swine Nutrition farm. All animals were humanely euthanized by captive bolt and exsanguination.

#### 5 or 32 day sensitization periods

Pigs were sensitized via intradermal injection with either 1mL 10% 2,4-dinitrofluorobenzene (DNFB, Sigma Aldrich cat. D1529) in vehicle [14], 1 mL 1% oxazolone (OXA) in vehicle [13], or 1 mL 1:1 acetone:olive oil vehicle; all solutions were administered by ten 0.1 mL intradermal injections across the back (for a total volume of 1mL) (S1A Fig). After a sensitization period of 5 or 32 days, animals were challenged re-exposed with 0.1 mL 1% OXA, 0.1 mL 1% DNFB, and 0.1 mL vehicle via intradermal injection in separate dorsal ear locations (two injection sites per ear). We measured ear thickness at 24, 48, and 72 hours post challenge using skinfold calipers (Harpenden, England, UK).

#### 7 or 21 day sensitization periods

Pigs were sensitized by adding 0.2 mL of solution dropwise to the pig’s shaved back between their shoulder blades (“painting”) (S1B Fig) with either 2.5% DNFB (Sigma-Aldrich Cat # D1529), 10% DNFB, or 4:1 acetone:olive oil vehicle which in preliminary comparisons to 1:1 vehicle was faster drying (original vehicle from [13]). After a sensitization period of 7 or 21 days, animals were challenged with 0.2 mL 1% DNFB, 1% OXA (Sigma-Aldrich Cat # E0753), or vehicle via intradermal injection in separate dorsal ear locations (one injection site per ear). We measured ear thickness at 24, and 48 hours post challenge using skinfold calipers.

Treatment groups are designated by (sensitization treatment)/(challenge treatment) where D = DNFB treatment; O = OXA treatment; & V = vehicle treatment. For example, an animal sensitized with vehicle and challenged with DNFB is designated V/D.

### Ear punch collection and histology scoring

Upon euthanasia, ears were removed and biopsy punches (Fisher, cat. # 475.20040.2) were collected at site of challenge injection. Ear punch biopsies were fixed in 3.7% formaldehyde in 1X PBS and then transitioned to 70% ethanol after 24 hours and were maintained at 4°C. The tissues were routinely processed to slides and stained with hematoxylin and eosin by the USDA National Animal Disease Center (NADC) Microscopy Service Team. A USDA pathologist, who was blinded to the treatment groups, evaluated and scored the slides.

### Ear punch section CD3 immunohistochemistry

Ear sections were deparaffinized and rehydrated using the Leica ST5020. Samples underwent heat-induced epitope retrieval, in sodium citrate, pH 6.0 at 95ºC for 20 minutes. The sections were then blocked for non-specific enzymatic reactions with Dual Endogenous Enzyme Block (cat. # S2003, Dako) for 10 minutes at room temperature. This was followed by blocking for non-specific protein interactions with Protein Block Serum-free (cat. # X0909, Dako) for 20 minutes at room temperature. Subsequent staining and detection with polyclonal rabbit anti-human CD3 (cat. # A0452, Dako) for 1 hour, followed by Dako EnVision+ system-HRP anti-rabbit polymer (cat. # K4002, Dako) for 30 minutes was performed. DAKO® Liquid DAB (3,3′diaminobenzidine tetrahydrochloride) + Substrate-Chromogen System (cat. # K3467, Dako) was used for 3 minutes, then counterstained for 1 minute with Gills hematoxylin for visualization of antigen-antibody reactions. Sections were then dehydrated on the Leica ST5020 Multistainer and coverslipped using a Leica CV5030. A USDA pathologist, who was blinded to the treatment groups, evaluated and scored the slides.

### Statistical analysis

#### Statistical analysis of pathology ear scores

Statistical analysis of pathology scores were conducted using a multiple linear regression model in R [18]. Fixed effects of sensitization period (7 or 21 days) and previous hapten exposure (vehicle sensitized/ DNFB challenged (VD) and DNFB sensitized/ DNFB challenged (DD) groups) were fit, and if appropriate, the interaction of sensitization x treatment was additionally included in the model. The LSmeans and contrast differences between VD and DD groups are reported. Statistical significance was declared when the *p-*value ≤ 0.05.

#### Statistical analysis of ear thickness change data

Cohorts were analyzed with R [18]. Basic least-squares fixed-effects models were fit for all cohorts (using the lm function equivalent to PROC GLM from SAS). The following models were run for each of the four cohorts with the number of levels given in parentheses. Ear thickness at 24 and 48 hours was evaluated. Type III ANOVA tests were used for all effects. LSmeans and associated contrasts (i.e. differences in LSmeans) were calculated from the lsmeans package in R [19].

### 5, 32 day Model

Ear Thickness (increase from time 0) =

trial + sex + ear location + challenge treatment + sensitization treatment + challenge treatment*sensitization treatment + error

Trial was the effect of the two separate groups of pigs for each the 5 and 32 day sensitization lengths; sex was the effect of either castrated males or female pigs; ear location was the effect of each location A, B, C, or D (2 on each ear); challenge treatment was the effect from no injection, vehicle, OXA, or DNFB injection in the ear; sensitization treatment was the effect from either no injection, vehicle, OXA, or DNFB injection on the back.

### 21 day Model

Ear Thickness (increase from time 0) =

Trial + Sex + challenge treatment + sensitization treatment + challenge veh + challenge method + challenge treatment*sensitization treatment + challenge treatment*challenge method + error

Trial and sex are defined above; challenge treatment was the effect of either vehicle or DNFB; sensitization treatment was the effect of either vehicle, low 2.5% DNFB, or high 10% DNFB painted on the back; challenge vehicle is the effect of 4:1 or 1:1 acetone:olive oil; and challenge method was the effect of injection or painting on the ear.

### 7 day Model

Ear Thickness (increase from time 0) =

trial + sex + challenge treatment + sensitization treatment +

challenge treatment*sensitization treatment + error

Trial and sex are defined above; challenge treatment was the effect of either vehicle, OXA, or DNFB injection in the ear; sensitization treatment was the effect of either vehicle or 10% DNFB painted on the back.

## Results

**Fig 1:**
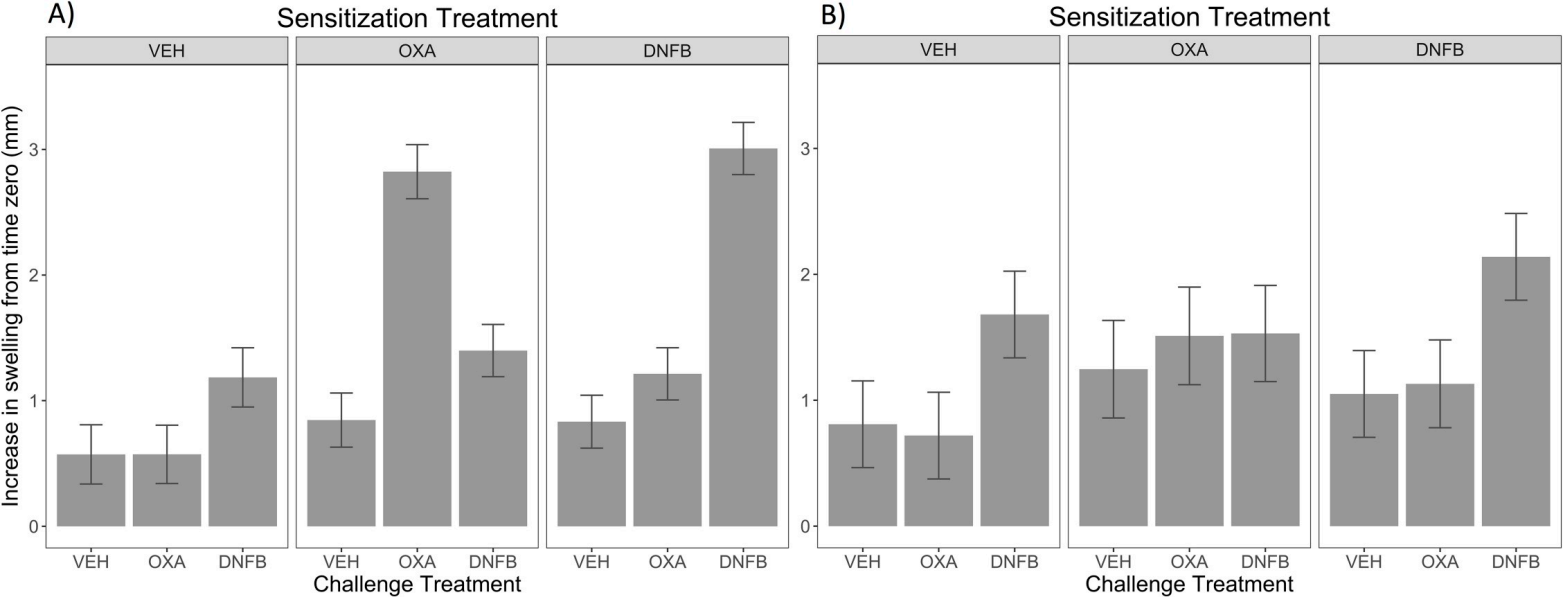
Ear thickness shows hapten-specific ear inflammation response in ears of pigs for 32 day sensitization but not for 5 day. Pigs were sensitized by intradermal injection on the back with DNFB, OXA, or 1:1 acetone:olive oil vehicle alone as indicated at the top of each graph. Animals were challenged by intradermal injection in the ear either A)32 or B)5 days after sensitization with the indicated ear treatment. Data shown is from the 48 hour time point, and is reported as the LS means difference in ear thickness (mm) from time zero, pre-sensitization. The interaction of back sensitization*ear treatment was evaluated for significance to provide evidence for hapten-specific memory. With a threshold p-value <0.05, 32 day (p-value < 0.01) sensitization period showed evidence of hapten-specific ear swelling, while the 5 day sensitization period (p-value < 0.98) did not. Error bars represent standard error.

**Fig 2:**
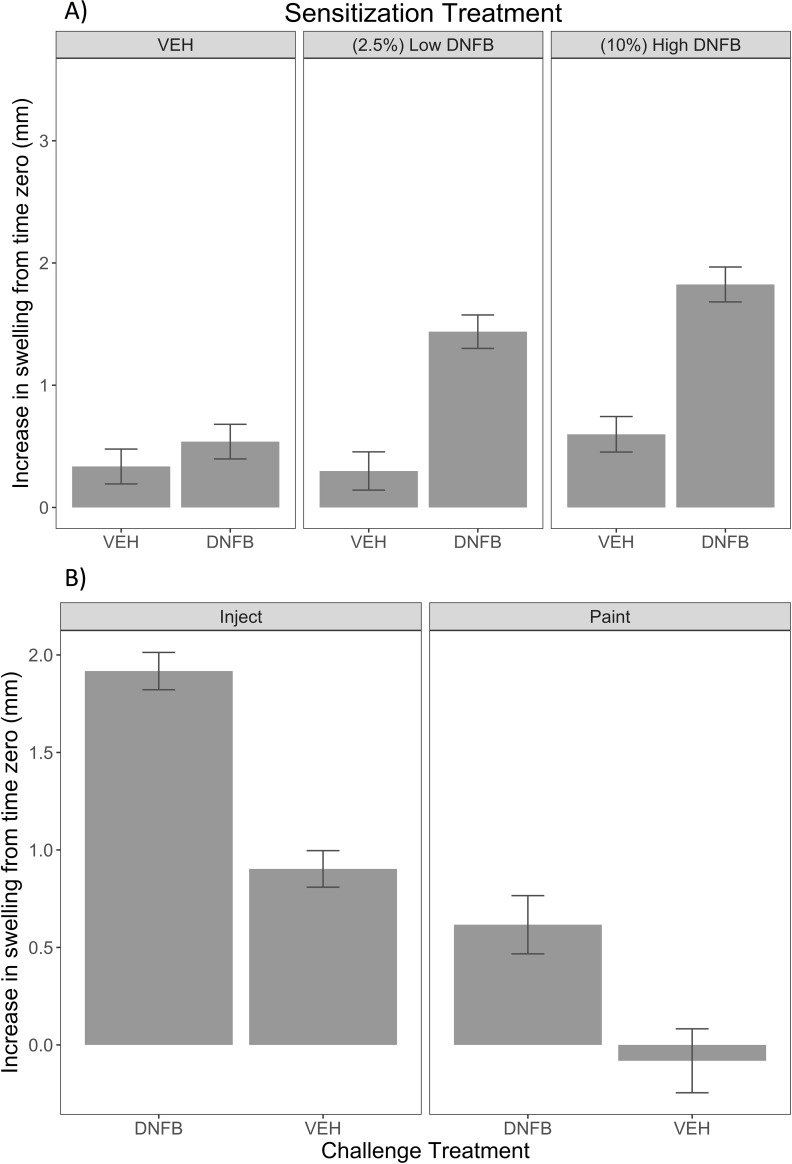
Improvements to CHS protocol shows topical application of sensitizing agent causes less background inflammatory response with 21 day sensitization period. (A) Dosage comparison for sensitizing concentration of topical dorsal application (paint) of DNFB low (2.5%) versus DNFB high (10%). (B) Ear challenge methodology comparison between injection vs topical painting application across both sensitization concentrations after 48 hours post challenge. Error bars represent standard error.

**Fig 3:**
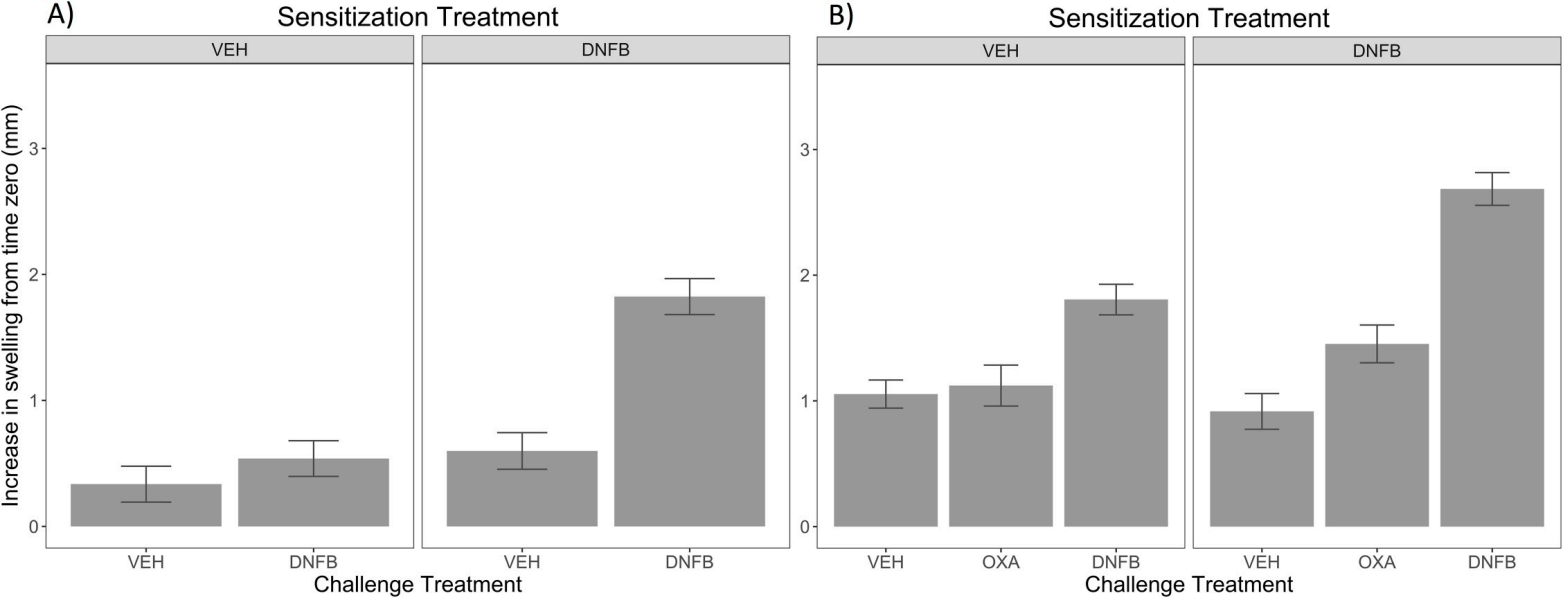
Improved protocol ear thickness shows hapten-specific ear inflammation response in ears of normal pigs as early as 7 days post-sensitization. 15–30 kg pigs had DNFB or 4:1 acetone:olive oil vehicle applied dropwise to their backs. Animals were challenged after 21 or 7 days with intradermal ear injections of DNFB, OXA, or respective vehicle. Shown here is the LS means of ear thickness difference (mm) from time zero to 48 hours for sensitization periods of (A) 21 days or (B) 7 days. The interaction of back sensitization*ear treatments was evaluated for significance of hapten-specific memory. With a threshold p-value <0.05, 21 day (p-value < 0.01) and 7 day (p-value< 0.01) sensitization periods showed evidence of hapten-specific ear swelling. Error bars represent standard error.

This research sought to establish whether porcine immunity can create hapten-specific CHS memory responses in swine and to develop a useful large animal model to study specific memory responses. CHS response was measured as the increase in ear thickness from time zero (challenge) to the post-challenge time. We also determined whether such responses were hapten-specific, i.e., were higher for ear locations challenged with the chemical for which the animal had been sensitized as compared to other treatments (**Figs 1–3**). The significance of differences in response between hapten was determined by evaluating the interaction of each ear treatment (challenge) with each back treatment (sensitization).

We integrated protocols that had been used to demonstrate CHS in pigs [13, 14] with those used in mice to demonstrate innate memory of NK cells to prior haptens [2, 9]. Initially, we repeated the protocols that topically applied haptens to the skin of the inner thigh [14], but found it difficult to maintain a localized effect, and without slings, to keep the area clean. Instead, we performed intradermal injections on the dorsal back over ten locations more similar to other sensitization work reported [13](see S1 Fig). Hapten challenge was then performed with four ear injections (two per ear) of DNFB, OXA, and vehicle controls, and changes in ear thickness was measured at 24, 48 and 72 hours post-challenge. Results demonstrated hapten-specific ear swelling for DNFB and OXA was statistically significant for a sensitization period of 32 days (*p*-value < 0.01) as seen in **Fig 1A**. However, when the same protocol was utilized with a 5-day sensitization to approximate work in the mouse literature, no evidence of hapten-specific swelling was detectable (*p*-value 0.98, **Fig 1B**).

Further, in an attempt to reduce the number of animals required for a trial, we elected to use two challenge injections per ear, reasoning that the CHS effect should be local and this design would decrease the number of animals needed for the studies. However, visual cues of ear irritation/inflammation throughout the ear post-challenge in such studies led us to statistically evaluate whether one ear treatment injection could affect the other challenge injection in the same ear. The effect of ear injection location (whether the injection site was proximal or distal to the pig’s head) was found to be a significant fixed effect for ear swelling with a *p*-value <0.01 for the 5 day sensitization period but this effect was not significant for 32 day sensitization (*p*-value = 0.72). Therefore, to improve our protocol we adjusted numerous components of our methodology.

First, to avoid any potential within-ear interactions across challenge treatments (DNFB versus OXA), only one challenge injection was applied per ear. As this increased the number of animals required for these studies, we focused on DNFB sensitization alone in these optimizations. Second, we tested several adjustments to the dorsal sensitization protocol. To decrease the time required for resolution of inflammation seen in the intradermal hapten injections, we tested a topical application of sensitizing agents on the cranial back of the pig, which required a faster drying vehicle. We found that 4:1 acetone:olive oil was substantially faster drying than the 1:1 acetone:olive oil [13] we used previously. Third, to determine if we could reduce the concentration of DNFB used in sensitization, we tested two topical concentrations of DNFB 2.5% or 10% (0.2 mL) over a 21 day sensitization period as shown in **Fig 2A**. While ear thickness response to hapten sensitization was detected in both high and low concentrations of DNFB (*p*-value < 0.01), the effect was larger for the high concentration of DNFB (LSmeans of 1.82 mm vs 1.44 mm, *p*-value = 0.04). When we accounted for vehicle background (differences between DNFB sensitized/DNFB challenged (DD) and vehicle sensitized/DNFB challenged (VD)), the 10% DNFB dosages also had a larger effect; 1.27 ± 0.19 mm for 10% and 0.88 ± 0.19 mm for 2.5%. Fourth, we also tested if application by topical painting onto the pig ears could be used for challenge, rather than intradermal injection (**Fig 2B**). The difference in LSmeans for change in thickness effect size for challenge treatments between vehicle and DNFB was similar, but showed increased swelling for the injection method (1.0 mm for injection and 0.7 mm for topical painting). We also noted a larger vehicle effect with injections, which is not desired. Finally, we observed operationally that a single well-defined injection site allowed for more accurate measuring of ear thickness compared to the larger surface area affected by painting in pig ears which vary greatly in thickness throughout the ear. We chose to move forward with topical sensitization with 10% DNFB on the dorsal back and intradermal injection ear challenge methods, as shown in **Fig 2** for a day 21 sensitization trial with DNFB alone.

To determine if pigs could develop hapten-specific memory, we used the new protocol adjustments illustrated in **Fig 2** (10% DNFB painting sensitization and single ear injection challenge) and evaluated a 21-day sensitization, and a 7-day sensitization period (**Fig 3**). The effect of prior sensitization was significant for specific challenges as measured by the interaction of challenge treatment*sensitization treatment for 21 days (**Fig 3A**) *p*-value < 0.01. Significant differences in ear thickness were observed between vehicle sensitized/DNFB challenged (VD) and DNFB sensitized/DNFB challenged (DD) groups (DD ear measurements higher) for both 24 and 48 hours post challenge, indicating that the inflammatory response was significantly greater when the pig had been exposed previously to the haptens. We then tested a shorter sensitization period, and found similar significant effects of a 7-day prior sensitization of DNFB on subsequent DNFB or OXA challenge (**Fig 3B**) (*p*-value <0.01). **Table 1** shows statistical evidence (*p*-values) for significant differences in response to treatment combinations for specific contrasts.

**Table 1:** P-values of contrasts examining the effect of a sensitizing hapten. Reported are ANOVA *p*-values for the overall interaction of sensitization*ear treatment, followed by *p*-values of the hapten-specific contrasts at either 24 or 48 hours post challenge. Comparisons investigated the effect of sensitizing the pig with a hapten prior to hapten challenge. Primary comparisons included vehicle sensitized, DNFB challenged (VD) compared to DNFB sensitized, DNFB challenged (DD) and vehicle sensitized, OXA challenged (VO) compared to OXA sensitized, OXA challenged (OO). Also included is the contrast of OXA sensitized, DNFB challenged (OD) compared to DNFB sensitized, DNFB challenged (DD) and contrast of DNFB sensitized, OXA challenged (DO) compared to OXA sensitized, OXA challenged (OO) which explores the hapten-specific response of the inflammation.

Sensitizing Method	Sensitization Period (days)	Hours post challenge	ANOVA *p*-value back*ear interaction	VD vs DD (*p*-value)	OD vs DD (*p*-value)	VO vs OO (*p*-value)	DO vs OO (*p*-value)
Back injection	32	24	<0.01	<0.01	<0.01	<0.01	<0.01
		48	<0.01	<0.01	<0.01	<0.01	<0.01
	5	24	0.93	0.79	0.80	0.32	0.45
		48	0.61	0.34	0.23	0.13	0.47
Back topical paint	21	24	<0.01	<0.01	N/A	N/A	N/A
		48	<0.01	<0.01	N/A	N/A	N/A
	7	24	<0.01	<0.01	<0.01	N/A	N/A
		48	<0.01	<0.01	<0.01	N/A	N/A

**Fig 4:**
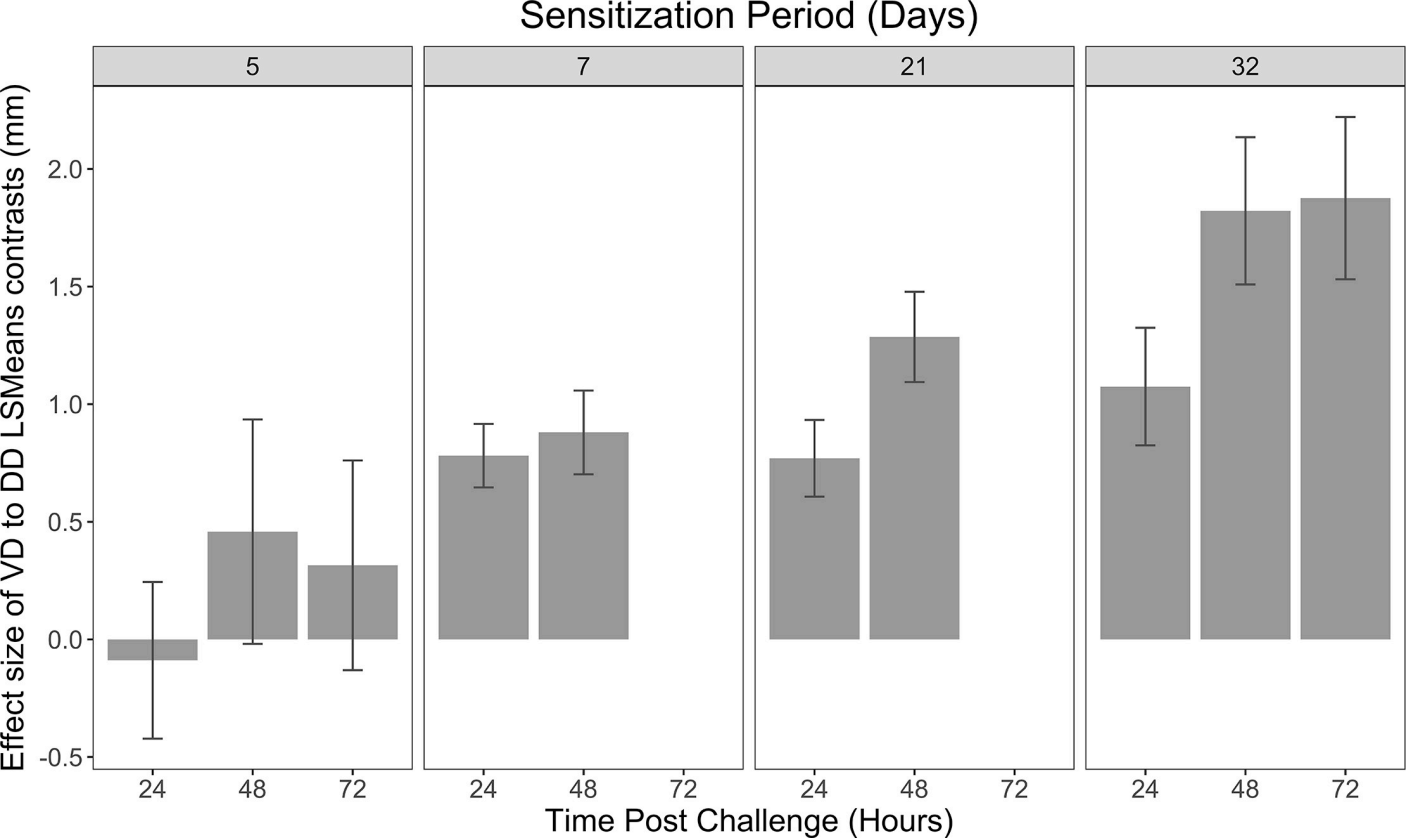
Hapten-specific ear swelling effect differences between sensitization periods and time of data collection. While 7, 21 and 32 day sensitization periods yielded significant hapten-specific inflammation responses, the 32 day sensitization period showed the largest effect of prior sensitization. Data shown for 24, 48, and 72 hour (if applicable) ear thickness. Error bars represent standard error.

To directly address the effect of prior sensitization, we compared the difference in LSmeans in ear thickness change between the VD and DD treated animals across sensitization period (5, 7, 21, 32 days) and at time of collection post challenge (24, 48, or 72 hours (only available for 32 and 5 day sensitization periods)) (**Fig 4**). The trend in difference in LSmeans increased with length of sensitization period (5 to 32 days). At day 5, contrasts were not significantly different from zero. All other longer sensitization periods led to statistically significant contrasts.

To provide further characterization of the hypersensitivity reaction induced in the model, post-mortem ear biopsy punches were collected at 48 hours post challenge for 21 and 7 day sensitization trials utilizing the modified CHS memory protocol. Sections of fixed tissue samples were scored for cellular infiltrates, vasculitis and thrombosis, and necrosis, with a rising score from 0–4 for each independent parameter (0 is absent, while 4 is completely diffuse). **Table 2** depicts average scores for four ear punches (1 ear punch per animal) per treatment (VD and DD) from the 7 and 21-day sensitization period (seen in **Fig 3**). DD ear punches had significantly higher cellular infiltrate scores (*p*-value < 0.01) (**Table 2**), than VD treated ears for both 7 and 21 day sensitization periods, but there was no statistical difference due to sensitization for thrombosis scores (*p*-value 0.39), or necrosis (*p*-value 0.18).

**Table 2:** Histology and immunohistochemistry scoring of ear sections from VD and DD hapten treated ear biopsy samples (7 and 21 day sensitization periods). Measures of pathology, including cellular infiltrate, vasculitis and thrombosis, and necrosis were scored from H&E preps on a 0–4 scale. In addition, CD3 immunostaining of ear biopsy samples was also performed and scored on a 0–4 scale. Depicted are the Lsmeans and standard errors of 4 ear punches per VD and DD treatment for both the 7 and 21 day sensitization studies and the *p*-value testing statistical significance of the difference between VD and DD groups within sensitization period.

7 Day Sensitization Period Pathology Scores (0–4)				
Treatment	Cellular Infiltrate	Vasculitis and Thrombosis	Necrosis	CD3 Immunostaining
Vehicle Sensitized, DNFB Challenge (VD)	1.00 ± 0.38	1.13 ± 0.49	1.81 ± 0.46	1.00 ± 0.40
DNFB Sensitized, DNFB Challenge (DD)	2.50 ± 0.38	1.63 ± 0.49	2.56 ± 0.46	1.38 ± 0.40
*p-*value	<0.01	0.39	0.18	0.52
21 Day Sensitization Period Pathology Scores (0–4)				
Treatment	Cellular Infiltrate	Vasculitis and Thrombosis	Necrosis	CD3 Immunostaining
Vehicle Sensitized, DNFB Challenge (VD)	1.13 ± 0.38	1.25 ± 0.49	2.31 ± 0.46	0.63 ± 0.40
DNFB Sensitized, DNFB Challenge (DD)	2.63 ± 0.38	1.75 ± 0.49	3.06 ± 0.46	2.88 ± 0.40
*p-*value	<0.01	0.39	0.18	<0.01

**Fig 5:**
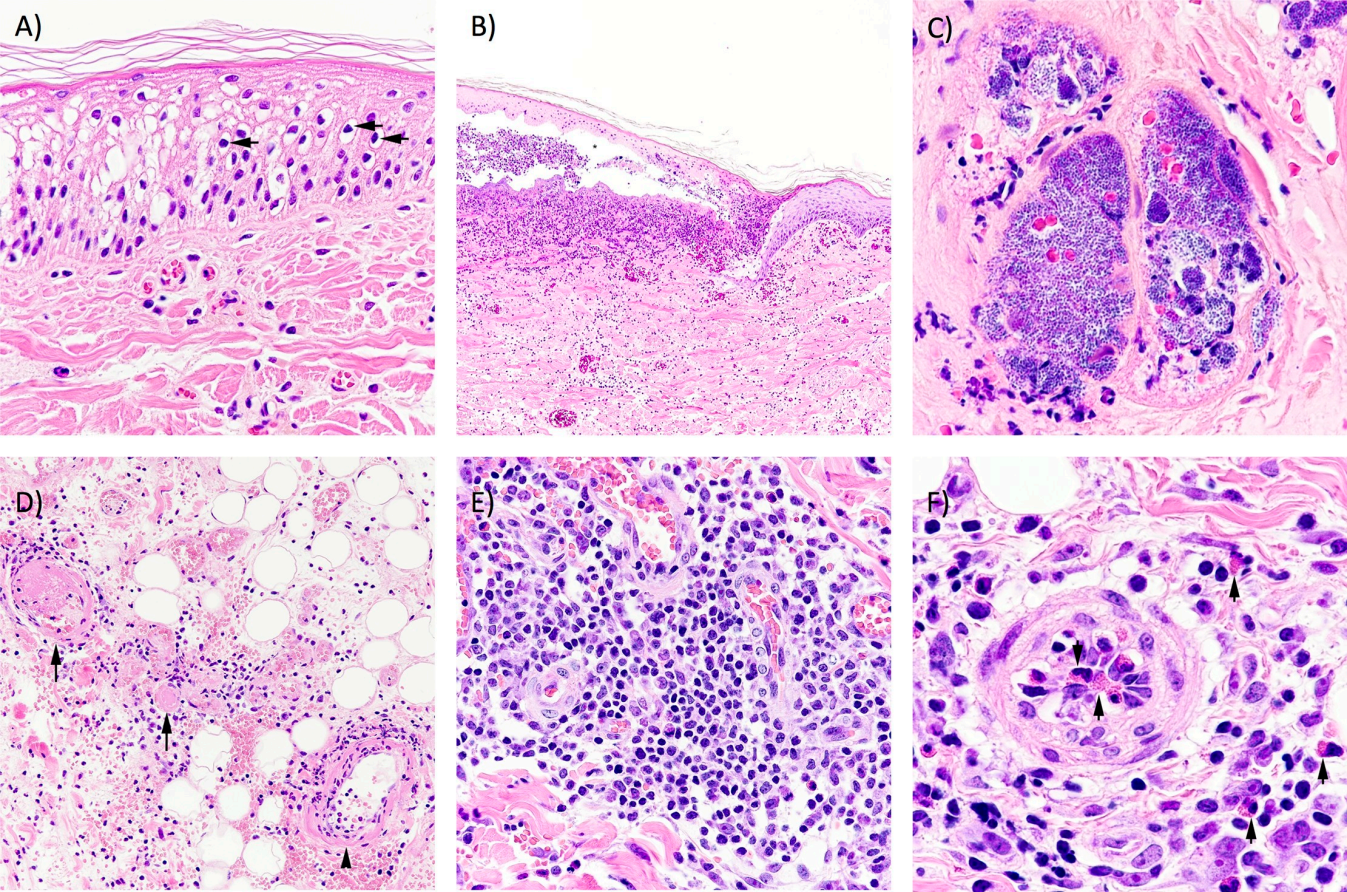
Haemotoxylin and Eosin Stained Histopathology of ear sections from VD and DD hapten treated animals (7 and 21 day sensitization period). A) Skin epidermis from a DD animal (7 day sensitization). Note the vacuolated epidermal cells and shrunken pyknotic nuclei (arrows). B) Skin epidermis from VD animal (7 day sensitization). Note the cleft within epidermal layers (*) containing neutrophils and fibrin. Basal epidermal layers and superficial dermis contain numerous inflammatory cells. C) Skin from DD animal (7 day sensitization) vessels within epidermis contain numerous basophilic coccobacilli. D) Subcutis skin from DD animal (21 day sensitization). Note the vessels containing fibrin thrombi (arrows), and vasculitis and fibrinoid degeneration (arrowhead). E) Dermis skin from DD animal (21 day sensitization). Vessels are surrounded by numerous inflammatory cells, mostly lymphocytes. F) Dermis skin from DD animal (7 day sensitization). Granulocytes are evident within perivascular infiltrate as well as within arteriole.

Histologically, microscopic changes were similar in pigs sampled at 7 and 21 days after hapten exposure; however, these changes differed in the degree of severity. Hapten challenge following either DNFB or vehicle sensitization resulted in some level of focal epidermal degeneration and necrosis involving all epidermal layers. Degenerative keratinocytes were characterized by vacuolar degeneration and rounded, hyperchromatic nuclei (**Fig 5A**). In necrotic areas there was loss of cellular detail with small hypochromatic, sometimes indiscernible nuclei. In some areas, between both VD and DD samples, superficial layers of epidermis were separated from deeper epidermal layers forming a cleft, the inside of which contained neutrophils and fibrin (**Fig 5B**). In 21 day samples greater numbers of neutrophils, as well as colonies of basophilic coccobacilli, were seen in more extensive areas of epidermal necrosis compared to 7 day samples for both VD and DD sections. For both sensitization time points the dermis and subcutis were greatly expanded due to edema and contained scattered infiltrates of lymphocytes and lesser numbers of granulocytes. For both VD and DD samples, colonies of basophilic coccobacilli were present, at times within blood vessels (**Fig 5C**). Occasional vessels contained fibrinous to fibrinocellular thrombi. Thrombosed vessels were most commonly found in vasculature adjacent to the perichondrium (**Fig 5D**). The epidermal surface opposite the hapten exposed area was normal; however, superficial and deep dermal vessels contained prominent plump endothelial cells and were variably surrounded by lymphocytic infiltrates (**Fig 5E**). Microscopically, samples from re-exposed pigs (DD treatment) demonstrated similar changes to those seen in VD groups; however, epidermal degeneration and necrosis were more extensive and more severe. Cellular infiltrates were predominantly perivascular and lymphocytic, although granulocytes were also a notable feature of the perivascular infiltrative cells (**Fig 5F**).

**Fig 6:**
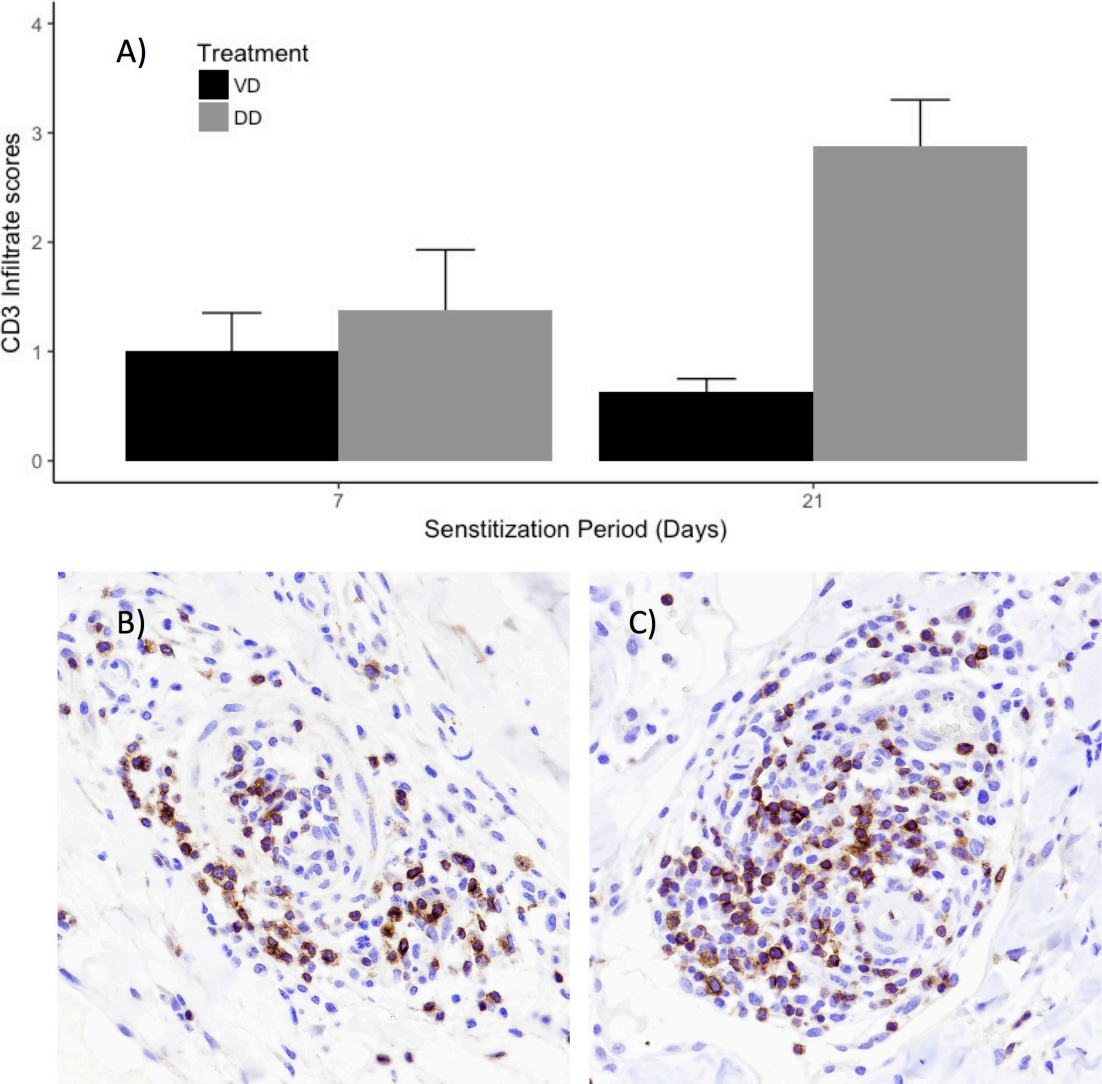
CD3 Immunohistochemistry staining and pathology scores of ear sections from VD and DD hapten treated animals (7 and 21 day sensitization period). A pathologist scored CD3 positive cell infiltrates on a 0–4 scale for VD (black bars) and DD (gray bars) for 7 and 21 day sensitization periods (A). Shown are representative images of CD3 stained DD ear sections from 7 day sensitization (B), and 21 day sensitization periods (C).

In addition to H&E slide scoring and analysis, immunostaining was completed for ear tissue sections to further characterize the immune cell response at the ear site of challenge. CD3 immunohistochemistry staining was completed for VD and DD ear biopsies, for 7 and 21 day sensitization periods (**Fig 6**), and scored by a pathologist on a 0–4 scale (**Table 2, Fig 6A**). While VD and DD CD3-positive infiltrates were not significantly different for the 7 day sensitization (**Table 2** *p*-value = 0.52), DD sections had significantly more CD3-positive cells at the site of challenge for the 21 day sensitization period (**Table 2**, *p*-value < 0.01). Within DD samples, DD sections from 7 day sensitization had significantly fewer CD3 stained infiltrates than DD sections from the 21 day sensitization period (**Fig 6B and 6C**) (*p*-value = 0.02), while VD sections were not statistically different between the two sensitization periods (*p*-value = 0.52).

## Discussion

The work herein described provides clear evidence of hapten-induced CHS memory for 7, 21, and 32-day sensitization periods. This accomplishes the primary objective of this work, which was to determine if hapten-specific CHS memory existed in a non-rodent model system. We also established and optimized a CHS protocol for measuring hapten-specific ear swelling inflammation responses in swine across a range of sensitization periods. For development of a porcine CHS memory protocol, we saw the largest effect of sensitization with 10% DNFB topically applied treatment, and an intradermal single-ear injection challenge treatment after 21 days.

It was important to establish we could successfully detect hapten-specific CHS memory after both long and short-term sensitization periods. While longer term, adaptive memory is well established, data from rodent models suggests that hapten-specific CHS can occur after 5 days from first exposure [2, 9]. Our initial protocol did not result in CHS with only a 5 day wait period between sensitization and challenge (5 day trial); however, our revised protocol did provide evidence of hapten-specific inflammation after a 7 day sensitization period.

Successful development of a CHS memory protocol in a large animal model using a shorter sensitization time may be important for testing innate memory functions, especially if immune compromised animal models are required. The possibility of innate memory training was supported in the current study by the hapten specific inflammation, despite the lack of differentiation in CD3-positive infiltrates between VD and DD groups during the short 7 day sensitization as compared to the 21 day sensitization period. Research has established that CHS innate NK cell memory is independent of the adaptive immune system. To illustrate this independence, rodent studies have utilized severe combined immunodeficiency (SCID) models [11], where memory responses are observed in T-B-NK+ SCIDs but are no longer present when using the non-functional NK cell [2] / or NK cell absent T-B-NK- SCID environments [9]. Numerous SCID pig models have emerged recently including models with NK cells present as well as NK cell absent (reviewed in [20]), thus the genetic tools, and now protocols, exist to demonstrate whether innate memory exists in swine. A short term sensitization period may also be critical in experiments using the established SCID pig model, who are susceptible to virtually all pathogens, and long term health can be difficult to achieve [21]. Genetically engineered SCID pigs have reported failure to thrive phenotypes and had to be euthanized after the first 30 days of life when reared in standard environments [22]. High health swine SCID colonies do exist but require significant care and facilities to rear long term SCID pigs successful [21]. The protocols developed herein may offer insight into the best sensitization time frame options for these valuable research models.

We show evidence of increased cellular infiltration in DD animals compared to VD pigs for both 7 and 21 sensitization periods (**Fig 5**), consistent with increased lymphocyte migration reported in the mouse studies [9]. However, the DD sections from the 21 day sensitization group had significantly more CD3 positive infiltrates compared to the DD sections from the 7 day sensitization; the latter was not significantly different from the VD sections from the 7 day sensitization (**Fig 6**). This is consistent with a CD3 T cell mediated memory response after a longer sensitization period. The increased infiltration of CD3 positives in the 21 day sensitization trial suggest researchers should carefully consider the type of immune cell response they wish to study, or avoid, when choosing sensitization periods.

We demonstrated the largest effects of previous hapten exposure with longer sensitization periods (**Fig 4**). Work with the lowest numbers of normal pigs possible may best utilize a longer sensitization period with the strongest power of detection as the effect size observed (VD vs DD in this example) is inversely proportional to the sample size and proportional to the power of the statistical test. Researchers addressing CHS memory questions in a porcine model should choose the most appropriate sensitization period for their experimental design.

In conclusion, we interpret these results as evidence that hapten-specific CHS memory exists in swine. Exploring immune memory is important for expanding our understanding of communication between the porcine adaptive and innate immune system training and education, which has implications for vaccine development in the commercial swine industry, as well as for human and veterinary biomedicine.

## Supporting information

S1 Fig Sensitization and challenge methods for showing contact hypersensitivity memory in swine. Commercial 15–30 kg pigs were utilized and individually housed throughout study. For sensitization periods of 5 or 32 days (A) animals were sensitized by intradermal injection on the back with DNFB, OXA, or vehicle alone. Pigs were challenged after 32 or 5 days with intradermal ear injections of DNFB, OXA, or respective vehicle. For sensitization periods of 7 or 21 days (B) animals were sensitized by ‘painting’ or adding solution dropwise on the back with DNFB or vehicle alone. Pigs were challenged after 7 or 21 days with intradermal ear injections of DNFB, OXA, or respective vehicle.
(TIFF)
