# Supplementary figures and images for: Successful development of methodology for detection of hapten-specific contact hypersensitivity (CHS) memory in swine
Source: PLoS One. 2019 Oct 9;14(10):e0223483. doi: 10.1371/journal.pone.0223483 (PMC6785115; doi:10.1371/journal.pone.0223483)

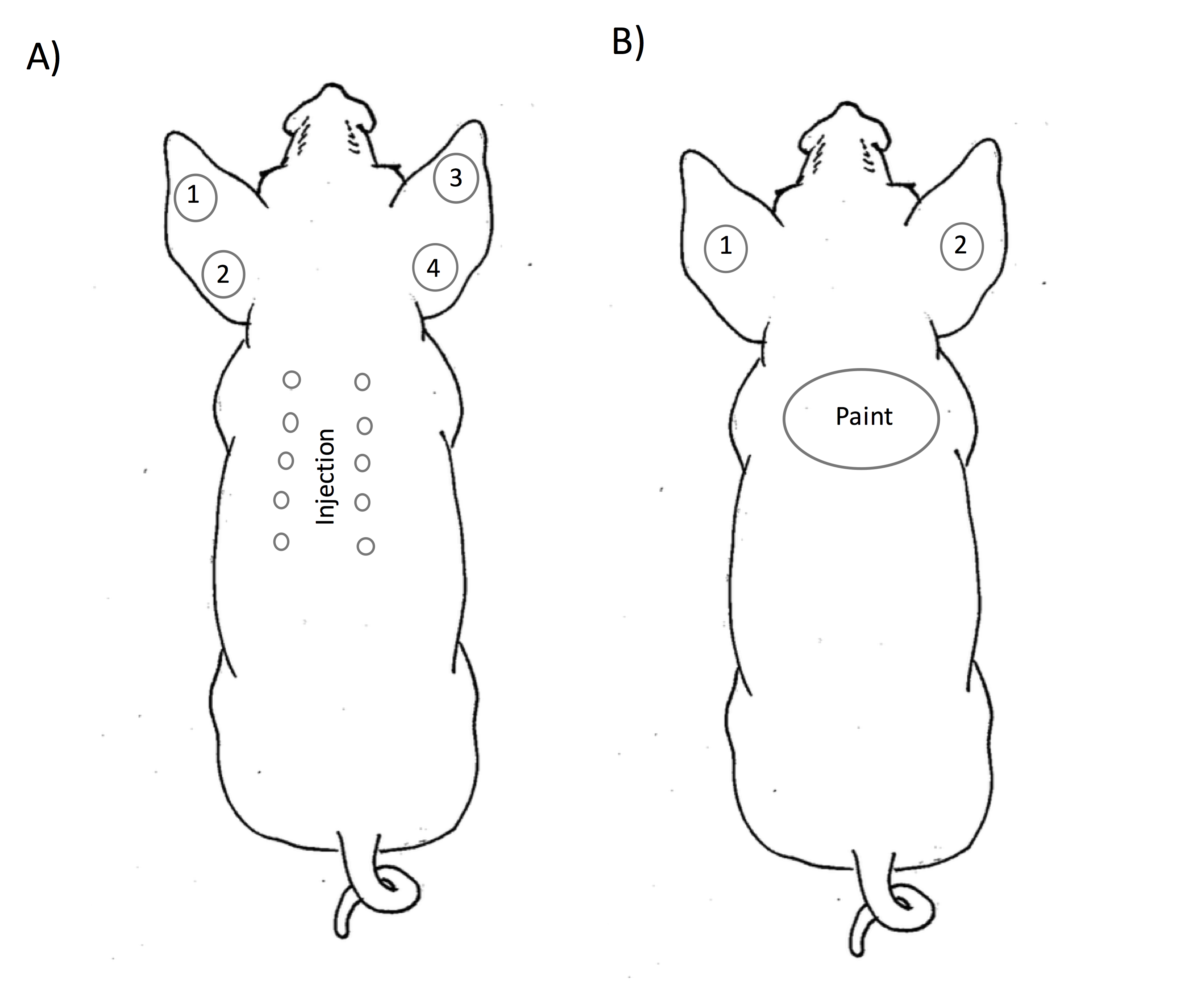

Supplement: S1 Fig — Commercial 15–30 kg pigs were utilized and individually housed throughout study. For sensitization periods of 5 or 32 days (A) animals were sensitized by intradermal injection on the back with DNFB, OXA, or vehicle alone. Pigs were challenged after 32 or 5 days with intradermal ear injections of DNFB, OXA, or respective vehicle. For sensitization periods of 7 or 21 days (B) animals were sensitized by ‘painting’ or adding solution dropwise on the back with DNFB or vehicle alone. Pigs were challenged after 7 or 21 days with intradermal ear injections of DNFB, OXA, or respective vehicle. (TIFF) [file pone.0223483.s001.tiff]
